# Gene Expression Profiling in Monocytes and SNP Association Suggest the Importance of the Gene for Osteoporosis in Both Chinese and Caucasians

**DOI:** 10.1359/jbmr.090724

**Published:** 2009-07-13

**Authors:** Xiang-Ding Chen, Peng Xiao, Shu-Feng Lei, Yao-Zhong Liu, Yan-Fang Guo, Fei-Yan Deng, Li-Jun Tan, Xue-Zhen Zhu, Fu-Rong Chen, Robert R Recker, Hong-Wen Deng

**Affiliations:** 1 Laboratory of Molecular and Statistical Genetics and Key Laboratory of Protein Chemistry and Developmental Biology of Ministry of Education, College of Life Sciences, Hunan Normal University Changsha, Hunan, Peoples Republic of China; 2 Osteoporosis Research Center and Department of Biomedical Sciences, Creighton University Medical Center Omaha, NE, USA; 3 Departments of Orthopedic Surgery and Basic Medical Sciences, University of Missouri–Kansas City Kansas City, MO, USA

**Keywords:** *STAT1*, bmd, monocytes, osteoporosis, microarray, SNP

## Abstract

Osteoporosis is characterized mainly by low bone mineral density (BMD). Many cytokines and chemokines have been related with bone metabolism. Monocytes in the immune system are important sources of cytokines and chemokines for bone metabolism. However, no study has investigated in vivo expression of a large number of various factors simultaneously in human monocytes underlying osteoporosis. This study explored the in vivo expression pattern of general cytokines, chemokines, and their receptor genes in human monocytes and validated the significant genes by qRT-PCR and genetic association analyses. Expression profilings were performed in monocyte samples from 26 Chinese and 20 Caucasian premenopausal women with discordant BMD. Genome-wide association analysis with BMD variation was conducted in 1000 unrelated Caucasians. We selected 168 cytokines, chemokines, osteoclast-related factors, and their receptor genes for analyses. Significantly, the signal transducer and activator of transcription 1 (*STAT1*) gene was upregulated in the low versus the high BMD groups in both Chinese and Caucasians. We also revealed a significant association of the *STAT1* gene with BMD variation in the 1000 Caucasians. Thus we conclude that the *STAT1* gene is important in human circulating monocytes in the etiology of osteoporosis. © 2010 American Society for Bone and Mineral Research.

## Introduction

Osteoporosis is mainly characterized by low bone mineral density (BMD). Genetic factors have important influences on BMD and osteoporosis.(1–3) Recent studies have shown that the immune system is strongly related to bone metabolism in terms of osteoimmunology.(4–8) Pathologic bone resorption was observed in immune system–related diseases such as autoimmune arthritis, periodontitis, Paget's disease, and bone tumors.(9)

Monocytes, important cells in immune system, produce a wide variety of factors such as interleukin 1 (IL-1), IL-6, tumor necrosis factor (TNF), transforming growth fator beta (TGF-β), and 1,25-dihydroxyvitamin D_3_ [1,25(OH)_2_D_3_]_._(10) These factors are involved in bone metabolism by regulating osteoclastic differentiation. Monocytes are also potential precursors of osteoclasts.(11,12) In vitro studies demonstrated that monocytes can differentiate into osteoclasts with bone resorption function.(13,14)

However, it is unknown whether other factors and mechanisms to regulate these factors are important in the ability of monocytes to affect bone metabolism. To address these questions, scientists have screened the differential gene expressions in osteoclastogenic cells using a high-throughput microarray platform.(15,16) Microarray technology has been used successfully for detection of gene expression profiles in diseases such as inflammatory breast cancer and urinary bladder cancer.(17,18) Theoretical studies also supported the reliability of using a microarray platform for the quantitative characterization of gene expression.(19,20) However, differential gene expression profiles in circulating monocytes associated with BMD variation had not been investigated until our previous research in Caucasian females.(16) In that study, we showed that chemokine receptor 3 (*CCR3*), histidine decarboxylase (*HDC*), and glucocorticoid receptor (*GCR*) genes in circulating monocytes potentially contributed to bone metabolism.(16)

The present study aims to identify significantly differentially expressed genes from 168 selected cytokine, chemokine, and osteoclastogenesis-related genes in circulating monocytes between the high and low BMD groups in Chinese Han females and validate the significant expression in Caucasian women. We also performed single-nucleotide polymorphism (SNP) association analysis with BMD to find further evidence of the identified genes at the DNA level.

## Materials and Methods

### Chinese subjects

The study was approved by the Research Administration Department of Hunan Normal University. Eight hundred and seventy-eight females who were Chinese Hans were recruited from Changsha City. All subjects signed informed-consent documents before entering the project. Healthy female subjects aged of 20 to 45 years were included because BMD reaches its peak and is most stable during this period. For each subject, we collected information on age, sex, medical history, family history, menstrual history, smoking history, physical activity, alcohol use, tea and coffee consumption, diet habits, etc. Female subjects must have regular menses to avoid the effects of menopause on BMD. Subjects with chronic diseases and conditions that potentially may affect bone mass have been excluded from the study. These diseases/conditions included chronic disorders involving vital organs (e.g., heart, lung, liver, kidney, and brain), serious metabolic diseases (e.g., diabetes, hypo- and hyperparathyroidism, and hyperthyroidism, etc.), skeletal diseases (e.g., Paget's disease, osteogenesis imperfecta, and rheumatoid arthritis, etc.), chronic use of drugs affecting bone metabolism (e.g., corticosteroid therapy and anticonvulsant drugs), and malnutrition conditions (e.g., chronic diarrhea, chronic ulcerative colitis, etc.). From the 100 top and 100 bottom hip BMD subjects we recruited all who consented to enter our potential future projects, including 14 high hip BMD (mean ± SD = 1.03 ± 0.05 g/cm^2^) subjects and 12 low hip BMD (mean ± SD = 0.7 ± 0.06 g/cm^2^) subjects (Table 1). Thirty milliliters of peripheral blood were drawn for each selected subject.

**Table 1:** Basic Characteristic Description of the Study Subjects in Chinese and Caucasians

	Female Chinese for gene expression		Female Caucasians for gene expression		Male Caucasians for SNP association		Female Caucasians for SNP association	
				
Trait	Low BMD(n = 12)	High BMD (n = 14)	Low BMD (n = 10)	High BMD (n = 10)	≤50 years (n = 250)	>50 years (n = 251)	Premenopausal (n = 249)	Postmenopausal (n = 250)
Age years	25.28 ± 3.14	28.67 ± 4.72	42.90 ± 1.91	41.70 ± 1.89	33.44 ± 9.66	67.33 ± 6.74	33.97 ± 8.45	66.36 ± 5.67
Height (cm)	158.88 ± 4.36	158.93 ± 5.28	160.46 ± 5.01	166.96 ± 7.30	180.00 ± 6.78	175.67 ± 6.63	165.38 ± 6.13	162.22 ± 6.43
Weight (kg)	51.54 ± 7.31	55.84 ± 5.73	58.00 ± 7.53	91.64 ± 19.58	88.03 ± 15.35	90.04 ± 14.47	70.74 ± 16.51	71.71 ± 5.10
Spine BMD (g/cm^2^)	0.85 ± 0.07	1.04 ± 0.09	0.90 ± 0.08	1.21 ± 0.08	1.05 ± 0.12	1.08 ± 0.20	1.05 ± 0.11	0.94 ± 0.10
Hip BMD (g/cm^2^)	0.70 ± 0.06	1.03 ± 0.05	0.79 ± 0.08	1.14 ± 0.09	1.07 ± 0.15	1.01 ± 0.14	0.95 ± 0.12	0.86 ± 0.14

*Note:* Values are the mean ± SD.

### BMD measurement

BMD (g/cm^2^) at the lumbar spine (L1–4, anteroposterior view) and total hip (femoral neck, trochanter, and intertrochanter region) was measured by a Hologic 4500-W dual-energy X-ray absorptiometry (DXA) (Hologic Corp., Waltham, MA, USA). The DXA scanner was calibrated daily, and long-term precision was monitored with external spine and hip phantoms. The coefficient of variation (CV) of measured BMD values was 0.80% at the hip.

### Monocyte isolation

**Fig. 1:**
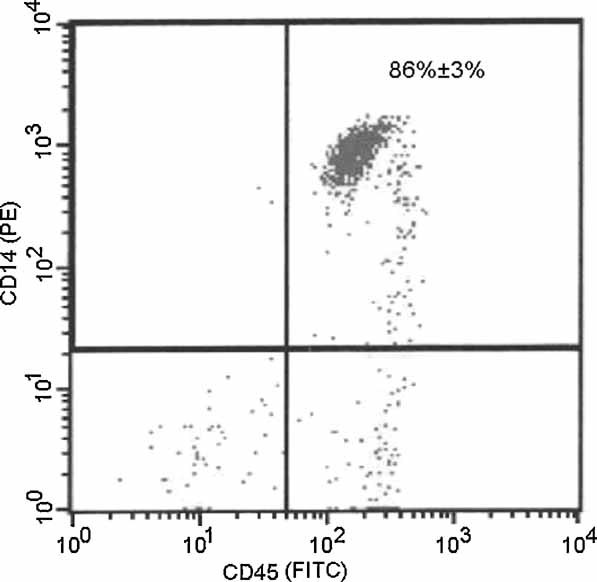
Flow cytometer analysis of the percentage of CD14^+^/CD45^+^ cells from human blood. CD14 and CD45 are the specific membrane markers on monocytes and mononuclear cells, respectively.

A monocyte negative isolation kit (Dynal Biotech, Inc., Lake Success, NY, USA) was used to isolate circulating monocytes from 30 mL of whole blood following the procedures recommended by the manufacturer. The kit contains a mixture of antibodies for CD2, CD7, CD16, CD19, CD56, and CD235a to deplete T cells, B cells, natural killer cells, erythrocytes, and granulocytes (if present), leaving monocytes untouched, pure, viable, and free of the surface-bound antibody and beads. Monocyte purity was assessed by flow cytometry (BD Biosciences, San Jose, CA, USA) with fluorescence-labeled antibodies PE-CD14 and FITC-CD45. The purity was 86% on average (Fig. 1).

### Total RNA extraction and microarray procedure

Total RNA from monocytes was extracted using a Qiagen RNeasy Mini Kit (Qiagen, Inc., Valencia, CA, USA). RNA integrity was assessed by using an Agilent 2100 Bioanalyzer (Agilent Technologies, Palo Alto, CA, USA). A total of 10 µg RNA from each sample was converted into biotinylated fragmented cRNA (BioArray HighYield RNA Transcription Labeling Kit, Enzo Diagnostics) that was hybridized (Affymetrix Genechip Hybridization Oven 640) to HG-U133 plus 2.0 GeneChip oligonucleotide arrays (Affymetrix, Santa Clara, CA, USA), which contains 54,675 sets of oligonucleotide probes that correspond to approximately 38,500 unique human genes, and then washed (Affymetrix Fluidics Station 450), stained with phycoerythrin-streptavidin, and scanned using Affymetrix Gene Array Scanner 3000.

### Statistical analysis

Transciptome-wide expression profiling involves a large number of genes and thus incorporates tremendous multiple tests. Some genes with suggestive significance may be excluded after the multiple-testing correction. If some genes are tightly related in a functionally relevant pathway, the *P* value for any single gene may not be significant after the multiple-testing correction. However, a focused expression screen on potential functional relevant genes largely can reduce the multiple tests and may increase the statistical power. Considering the multicomparison problem and the function of monocytes, 168 candidate genes were selected for statistical analyses. The 168 genes are all available cytokines, chemokines, osteoclast-related factors, and their receptors selected for our focused expression analyses of the Affymetrix HG133 plus 2.0 gene data set (see Appendix table). Microarray Suite 5.0 (MAS 5.0, Affymetrix) software was used to generate the array raw data files (CEL files). Then the probe-level data in CEL files were converted into expression measures and normalized by the robust multiarray average algorithm (RMA, http://www.bioconductor.org).(21) The differential expression analysis between low and high BMD samples was conducted by a nonparameter Wilcoxon signed-rank test. A Benjamini and Hochberg (BH) stepwise procedure was used for multiple-comparison adjustment,(22) and an adjusted *P* ≤ .05 was used as the significant criterion. Fisher's exact test was used in the canonical pathway analysis by Ingenuity Pathways Analysis (IPA) software (Ingenuity Systems, http://www.ingenuity.com) to test the association between genes within a canonical pathway and BMD variation. According to the similarity of gene expression, the differentially expressed genes were further analyzed for two-dimensional hierarchical clustering at both gene and sample levels.(23)

### Array replication in Caucasians

In this independent microarray study, we recruited 20 premenopausal Caucasian women, 10 with high BMD (spine or hip *Z*-score greater than +0.84) and 10 with low BMD (spine or hip *Z*-score less than −0.84; see Table 1) from the vicinity of Creighton University in Omaha, Nebraska, USA, for differential expression analyses in their circulating monocytes. Although the weights in the high BMD group were higher than in the low BMD group, no significant correlation of BMD with weight was detected in the low or high BMD group. This study was approved by the Institutional Review Board, and all the subjects signed informed-consent documents before entering the project. The inclusion and exclusion criteria were the same as in the Chinese population, but the age criterion was limited to the narrow range of 39 to 45 years, within the peak BMD range of 20 to 45 years. For the Caucasian samples, we used the Affymetrix HG-133A instead of the HG-U133 plus 2.0 that we used for the Chinese samples (the HG-133A chip contains fewer genes than the HG-U133 plus 2.0), but all the other experimental procedures and statistical analyses were the same as we described for Chinese samples.

### Validation by qRT-PCR in Caucasians

We used two-step qRT-PCR to confirm differentially expressed genes. Reverse-transcription reactions were performed in a 50 µL reaction volume containing 5 µL 10× PCR Buffer II, 11 µL 25 mM MgCl_2_, 10 µL dNTPs, 1.25 µL MULV reverse transcriptase, 1.0 µL RNase inhibitor, 2.5 µL Oligo d(T), 0.5 µg total RNA, and water to 50 µL. All these reagents were supplied by Applied Biosystems (Foster City, CA, USA). Reaction conditions were as follows: 10 minutes at 25°C, 30 minutes at 48°C, and 5 minutes at 95°C. Real-time quantitative PCR was performed in a 25 µL reaction volume using standard protocols on an Applied Biosystems 7900HT. Briefly, 2.5 µL of cDNA was mixed with 12.5 µL of TaqMan universal PCR master mix (2×), 1.25 µL of TaqMan gene express assay mix (contains forward and reverse primers and labeled probe), 1.25 µL of human GAPDH probe (20×), and 7.5 µL of water. The thermocycling conditions were as follows: 2 minutes at 50°C, 10 minutes at 95°C, and 40 cycles of 15 seconds at 95 °C plus 1 minute at 60°C. Based on the relative gene expression 2^−ΔΔ*Ct*^,(16) we performed Student's *t* test to confirm the differential expression genes. All reactions were run in triplicates for each gene.

### Confirmation of significant genes in SNP association study

#### Subjects and phenotype

For the association study, 1000 unrelated Caucasian subjects were identified from our established and expanding genetic repertoire, currently containing more than 6000 subjects. All subjects were U.S. Caucasians of European origin. The inclusion and exclusion criteria were the same as in the Chinese population for expression study, but males and postmenopausal women were included. The basic characteristics of all subjects are listed in Table 1. BMD values at spine and hip were measured using the Hologic 4500A DXA (Hologic, Inc., Bedford, MA, USA). The coefficient of variation of the DXA measurement was approximately 1.98% for spine BMD and 1.87% for hip BMD.

#### Genotyping and statistical analysis

Genomic DNA was extracted from whole human blood using a commercial isolation kit (Gentra Systems, Minneapolis, MN, USA). Genotyping with the Affymetrix Mapping 250K Nsp and 250K Sty arrays was performed. Fluorescence intensities were quantified using an Affymetrix Array Scanner 30007G. Data management and analyses were performed using the Affymetrix GeneChip Operating System. The final average Bayesian Robust Linear Model with Mahalanobis (BRLMM) call rate across the entire sample reached the high level of 99.14%. We tested the association of significant genes identified in the expression studies with BMD in the 1000 Caucasian subjects. Parameters such as age, age^2^, sex, age/age^2^-by-sex interaction, height, and weight were tested for their association with BMD at spine and hip. The significant (*P* ≤ .05) terms then were included as covariates to adjust the raw BMD values for subsequent analyses. Statistical analyses were performed using genotype and haplotype association software implemented in PLINK-1.03 (http://pngu.mgh.harvard.edu/purcell/plink/).(24) Linkage disequilibrium (LD) patterns were analyzed and plotted with the correlation coefficient between pairs of loci based on the 1000 unrelated Caucasians using the the Haploview program (http://www.broad.mit.edu/mpg/haploview/),(25) which describes more or less combinations of alleles. The haplotype block was used to show chromosome regions with high LD and low haplotype diversity in haplotpye association studies. MAPPER was used for searching transcript factor binding sites in the JASPAR database (http://mapper.chip.org/).(26)

## Results

The basic characteristics of the study subjects are shown in Table 1. Although hip BMD is the major study phenotype for the expression analyses in Chinese, spine BMD also was significantly different between the high and low BMD groups (*P* = 1.49 × 10^−6^). There were no significant differences in age and height traits between the high and low BMD groups for both Chinese and Caucasians for expression analyses. However, the weight and body mass index (BMI, kg/m^2^) in the low BMD group were significantly lower than in the high BMD group in Caucasians. We performed a general linear regression analyses for *STAT1* expression values and BMD status and incorporated weight and height as covariates in Caucasians. However, weight and height are not significant as covariates for *STAT1* expression analysis in the high and low BMD groups (*P* = .2529 and .2045, respectively). This implied that the weight and height in current study were not confounding factors for BMD in our expression analyses.

**Fig. 2:**
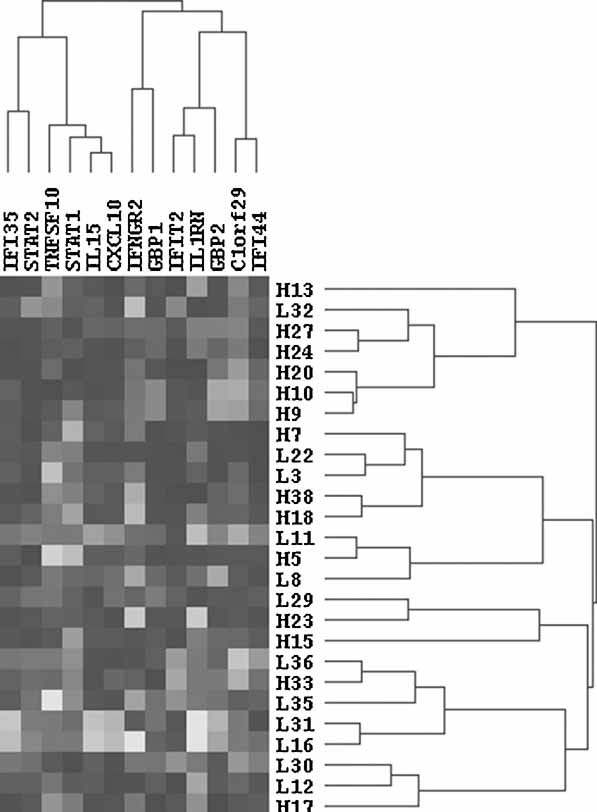
Two-dimensional hierarchical dendrograms clustered both the rows and columns of the data, the vertical axis showing the clustering of the subjects with different BMD (“L” for the low BMD and “H” for the high BMD) and the horizontal axis showing the clustering of the intensity of different gene expressions.

All the nominally significant genes (*P* < .05) of differential expression with BMD in Chinese are summarized in Table 2. We submitted our gene expression profiling to the Gene Expression Omnibus (http://www.ncbi.nlm.nih.gov/geo/), and the access number was GSE7158. After Benjamini and Hochberg correction for multiple comparisons, the differential expressions of signal transducer and activator of transcription 1 (*STAT1*) (adjusted *P* = .02248) and guanylate binding protein 1 (*GBP1*) (adjusted *P* = .03372) genes were still significant between the low and the high BMD groups. Expression fold changes of the significant genes were not large in the present study. The main reason might be that the female subjects in this study all were 20 to 45 years of age and with regular menses, in contrast with including both premenopausal and postmenopausal women in our previous study.(16) Actually, a 1.5-fold change has been shown to be significant in other differential gene expression studies.(27–30) In addition, BMD is a complex trait, and genes regulating its variation are not expected to have large differential expressions. In Fig. 2, a two-dimensional hierarchical dendrogram (based on the nominally significant genes listed in Table 2) shows the results of the hierarchical clustering analyses. Low BMD subjects were mainly clustered to the bottom of the figure.

**Table 2:** Differential Expression of Cytokine, Chemokines, and Osteoclastogenesis-Related Genes in Blood Monocytes From the Low and the High BMD Groups in Chinese

Probe ID	Gene symbol	Gene title	L-BMD intensity	H-BMD intensity	Fold L/H	Raw *P* value	Adjusted *P* value
200887_s_at	STAT1	Signal transducer and activator of transcription 1	857.21	582.34	1.47	.00008	.02248^*^
202269_x_at	GBP1	Guanylate binding protein 1	401.11	245.99	1.63	.00024	.03372^*^
204533_at	CXCL10	Chemokine (C-X-C motif) ligand 10	162.43	83.22	1.95	.00155	.062623
205992_s_at	IL15	Interleukin 15	234.54	174.21	1.35	.00310	.096789
214453_s_at	IFI44	Interferon-induced protein 44	321.75	186.31	1.73	.00693	.13910
225636_at	STAT2	Signal transducer and activator of transcription 2	404.73	339.6	1.19	.00937	.14628
202688_at	TNFSF10	Tumor necrosis factor (ligand) superfamily member 10	1094.65	889.05	1.23	.01084	.16032
204439_at	IFI44L	Interferon-induced protein44-like	424.81	153.59	2.77	.01261	.16106
242907_at	GBP2	Guanylate binding protein 2	335.3	214.65	1.56	.01677	.18849
209417_s_at	IFI35	Interferon-induced protein 35	158.99	115.84	1.37	.02209	.23874
201642_at	IFNGR2	Interferon gamma receptor 2	831.32	811.55	1.02	.02882	.27926
212657_s_at	IL1RN	Interleukin 1 receptor antagonist	468.82	256.04	1.83	.03721	.30425
226757_at	IFIT2	Interferon-induced protein with tetratricopeptide epeats 2	292.64	205.9	1.42	.03721	.30425

*Note:* Hybridization intensity and “present” status were given based on MAS5 algorithm. L-BMD intensity means hybridization intensity from the low BMD group; H-BMD intensity means hybridization intensity from the high BMD group. Fold L/H means the ratio of hybridization intensity from the low to that from the high BMD group. Raw *P* value means *P* value before multiple testing corrections. Adjusted *P* value represents *P* value adjusted with Benjamini/Hochberg method, and asterisks * means significant after Benjamini-Hochberg correction considering 281 probes of selected genes.

Many genes in interferon (IFN) pathway were differentially expressed. *STAT1*, *GBP1*, interferon gamma receptor 2 (*IFNGR2*), signal transducer and activator of transcription 2 (*STAT2*), guanylate binding protein 2 (*GPB2*), chemokine (C-X-C motif) ligand 10 (*CXCL10*), interferon-induced protein 44 (*IFI44*), interferon-induced protein 44-like (*IFI44L*), tumor necrosis factor (ligand) superfamily member 10 (*TNFSF*10), interferon-induced protein 35 (*IFI35*), and interferon-induced protein with tetratricopeptide repeats 2 (*IFIT2*) genes had higher expression in the low BMD group. Furthermore, the canonical pathway analysis supported the importance of interferon signaling pathway mediated by the *STAT1* gene in determining BMD variation (*P* = 1.67 × 10^−9^).

In the interleukin system, interleukin 1 receptor antagonist (*IL1RN*) and interleukin 15 (*IL15*) genes were upregulated in the low BMD group compared with the high BMD group. However, *IL6* gene expression was not detected.

Interestingly, the differential expression of the *STAT1* gene in circulating monocytes was replicated in our ongoing comparison microarray study between the low and high BMD premenopausal Caucasian women. Consistently, upregulation of the *STAT1* gene in the low BMD group also was significant after Benjamini and Hochberg correction for multiple testing (*P* = .0028, adjusted *P* = .048) (Table 3). qRT-PCR confirmed the significant differential expression of the *STAT1* gene in Caucasians (*P* = .0046) (see Table 3). For the *GBP1* gene, we did not find any significant results in both array and qRT-PCR analyses in Caucasians.

**Table 3:** Microarray and qRT-PCR Results for the Expression of *STAT1* Gene in Circulating Monocytes Between the Low and High BMD Groups in Chinese and in Caucasians

Population	Strategy	Expression value in low BMD	Expression value in high BMD	Fold change (L/H)	*P* value
Chinese	Affymetrix Microarray HG-U133 Plus 2.0	857.21 ± 292.94	582.34 ± 145.46	1.47	.02248*
Caucasians	Affymetrix Microarray HG-133A	957.18 ± 361.28	575.67 ± 243.00	1.66	.048*
Caucasians	qRT-PCR	3.13 ± 0.84	1.93 ± 0.75	1.62	.0046

*Note:* Expression value of Affymetrix Microarray was the hybridization intensity based on the MAS5 algorithm; expression value of qRT-PCR was relative quantity based on 2^−ΔΔ*Ct*^; fold L/H means the ratio of gene expression from the low to that from the high BMD group; * indicates the BH-adjusted *P* values for multiple testing.

**Fig. 3:**
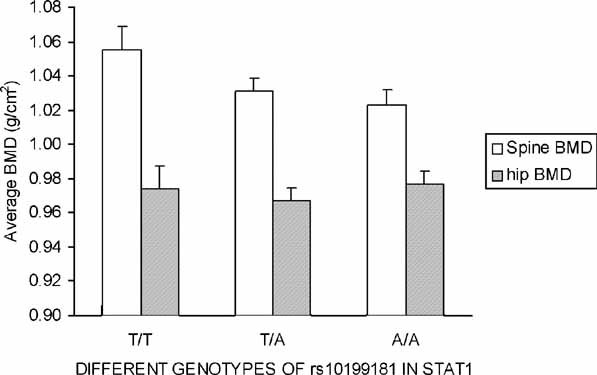
BMD (mean ± SE) in different genotypes of rs10199181 in the *STAT1* gene in Caucasians.

**Fig. 4:**
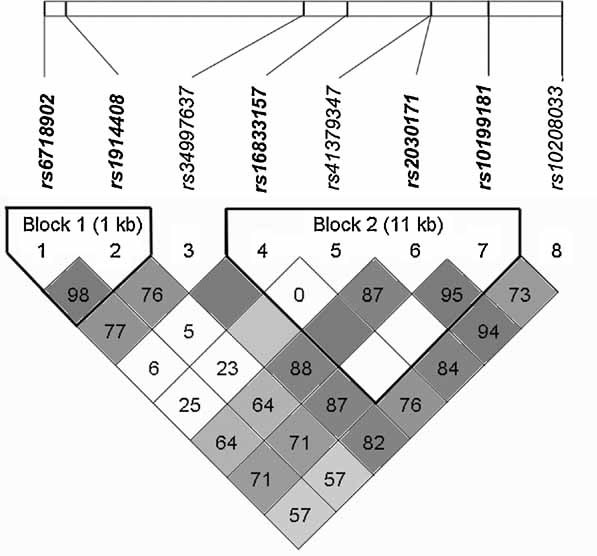
Pairwise linkage disequilibrium pattern of eight SNPs in the *STAT1* gene in 1000 unrelated Caucasians. Numbers in the squares are 100 by correlation coefficients (100*r*^2^) between pairs of SNPs. The intensity of shading is proportional to *r*^2^. SNP IDs in bold represent tag SNPs. Numbers in parentheses indicate lengths of haplotype blocks.

In SNP genotyping analysis using an additive model, two SNPs, rs10199181 (*P* = .0028) and rs2030171 (*P* = .0264), in the *STAT1* gene were associated with spine BMD (Table 4). It was obvious that subjects with the T allele of rs10199181 possessed high spine BMD (Fig. 3). Figure 4 shows the correlation coefficient between pairs of SNPs of the *STAT1* gene and reconstructed haplotype blocks. Interestingly, rs10199181 and rs2030171 were located in the same block, and a haplotype composed of SNPs rs16833157-rs2030171-rs10199181 (“G-G-A”) in the block also was demonstrated to be significantly associated with spine BMD (*P* = .0029). However, no significant association was detected for 14 SNPs in *GBP1* with BMD in Caucasians.

**Table 4:** Results for Eight SNP Association Analyses of the *STAT1* Gene for Hip and Spine BMD in Caucasians

SNP name	SNP ID	Position	Function	Allele^a^	*P* HWE^b^	MAF^c^	MAF^d^	*P* value (hip BMD)	*P* value (spine BMD)
SNP_A-1830221	rs6718902	191546449	Intron 24	A/G	0.7202	0.237	0.229	.1171	.2828
SNP_A-1966285	rs1914408	191548221	Intron 23	A/G	1	0.235	0.225	.1103	.2447
SNP_A-4228696	rs34997637	191567075	Intron 10	G/A	0.6512	0.234	0.250	.7317	.0895
SNP_A-2224968	rs16833157	191570643	Intron 9	A/G	0.3554	0.056	0.034	.5069	.0672
SNP_A-1966287	rs41379347	191577187	Intron 5	G/A	1	0.008	0.025	N/A	N/A
SNP_A-1966288	rs2030171	191577408	Intron 5	A/G	0.6562	0.321	0.300	.5866	.0264
SNP_A-4257270	rs10199181	191581798	Intron 4	T/A	0.7278	0.368	0.342	.2602	.0028
SNP_A-1783099	rs10208033	191587662	5' near gene	G/A	0.6373	0.400	0.450	.8659	.5721

N/A = The locus was not calculated for the significant test because of minor allele frequency of < 0.05.

a The former allele represents the minor allele of each locus.

b *P* value for Hardy-Weinberg equilibrium test.

c Minor allele frequency calculated in our Caucasian sample.

d Minor allele frequency reported for Caucasians in the public database of HapMap CEU.

## Discussion

In this study we investigated expression of 168 genes related to cytokines, chemokines, osteoclast formation factors, and corresponding receptors in monocytes from Chinese Han women with extremely discordant BMD. Thirteen genes were found to be differentially expressed. A very interesting phenomenon was that among the 13 genes, the *STAT1, IFI44L*, *CXCL10*, *IFI44*, *GPB1*, and *GPB2* genes were expressed in higher levels in the low BMD group than in the high BMD group, which is very similar to the IFN-induced gene expression pattern (IFN pathway). For instance, immature peripheral blood mononuclear phagocytes stimulated by the type I IFN isoform increased the expression of 44 genes, including *STAT1*, *IFI44L*, *CXCL10*, *IFI44*, *GPB1*, and *GPB2*.(31) Microarray analysis of cells infected with short hairpin RNA vectors pAB319 and pAB322 showed enhanced expression of many IFN target genes, such as *STAT1*, *IFI44L*, *CXCL10*, *IFI44*, and *GPB1*.(32) The increased expression of IFN pathway genes also was detected in blood mononuclear cells from patients with systemic lupus erythematosus and juvenile dermatomyositis(33) and in the human fibrosarcoma cell line.(34) The IFN pathway may regulate bone resorption in two ways. First, interferon-γ (IFNG) blocks *RANKL*-induced osteoclast differentiation.(9,35) Second, the IFN pathway in circulating monocytes may stimulate the secretion of cytokines IL-1, IL-6, and TNF to increase bone resorption.(36–38) In this study, the upregulation of *STAT1*-mediated IFN pathway genes in the low BMD group suggested the important effect of *STAT1* on bone resorption in vivo in humans.

For the 13 differentially expressed genes, however, only the *STAT1* and *GBP1* genes remained significant after correction for multiple testing. In our previous genome-wide array study on the same Chinese samples, we also found significant differential expression of the *STAT1* and *GBP1* genes in the array data analyses after correcting for multiple testing.(39) However, further qRT-PCR only confirmed the significance of the *GBP1* gene but not the *STAT1* gene.(39) Thus we tried to replicate significance of the two genes in our Caucasian expression study on circulating monocytes from 20 premenopausal Caucasian women (10 with low BMD and 10 with high BMD) and SNP association study on 1000 unrelated Caucasian subjects. We did not find the significance of the *GPBP1* gene in either replication study. Interestingly, however, the significance of the *STAT1* gene was found in both replication studies. In particular, significant upregulation of the *STAT1* gene was found in both array and qRT-PCR experiments in the Caucasian expression study. The *STAT1* gene was not differentially expressed in B cells isolated from peripheral blood between the high and low BMD subjects (data not shown) who were the same Caucasians for our current monocyte study. Therefore, it is likely that the alterations in *STAT1* expression only in monocytes, but not in other cells, are responsible for variations in bone mass in humans.

In the IFN signaling pathway, *STAT1* is a critical mediator gene.(9,40) In the above-mentioned IFN pathway for regulating bone resorption, *STAT1* mediates the effects of IFNG on both inhibition of *RANKL*-induced osteoclast differentiation(9,35,41) and secretion of IL-1, IL-6, and TNF.(36–38) In addition, in dexamethasone-treated peripheral blood mononuclear cell (PBMC) cultures, the inhibited IFNG expression suppressed expression of the *STAT1* gene.(42) Furthermore, in lupus nephritis patients, basal expression of *STAT1* was significantly higher in monocytes. and stimulation of the monocyte cultures with IFNG resulted in phosphorylation of *STAT1*.(43)

In mice, the *STAT1* gene plays an important role in bone metabolism in osteoblasts.(44) Recently, *STAT1* was reported to be upregulated in femur tissue in osteoporotic mice,(45) and this supports our finding of the high expression of *STAT1* in monocytes in human low BMD groups.

Interestingly, our published linkage study of BMD in 4126 human subjects also identified suggestive univariate and significant epistatic linkage signals at 2q32, which harbors the *STAT1* gene.(46) Furthermore, our group recently found significant linkage evidence on 2q32 with spine BMD using bivariate linkage analysis.(47) Our current SNP association study also replicated the significance of the *STAT1* gene for spine BMD in Caucasian samples. No SNP in the *STAT1* gene was associated with hip BMD at the SNP level, perhaps owing to different genetic determinants for spine BMD and hip BMD traits because many studies have shown different heritability and genetic loci underlying the two traits.(47,48) Hence our results tend to reveal the significance of *STAT1* on spine BMD. Subjects with the T allele of SNP rs10199181 in the *STAT1* gene tended to have a higher spine BMD than those with other alleles (see Fig. 3). According to the transcript factor Jaspar database, the T allele of rs10199181 is likely to bind transcript factor E4BP4, which might be induced by parathyroid hormone (PTH), a well-know hormone for bone growth, in osteoblasts.(49,50) The inducible effect of E4BP4 suggests a negative regulation by glucocorticoids that might decrease BMD.(51) Thus it implies that the T allele of rs10199181 in the *STAT1* gene may be involved in bone growth metabolism.

**Fig. 5:**
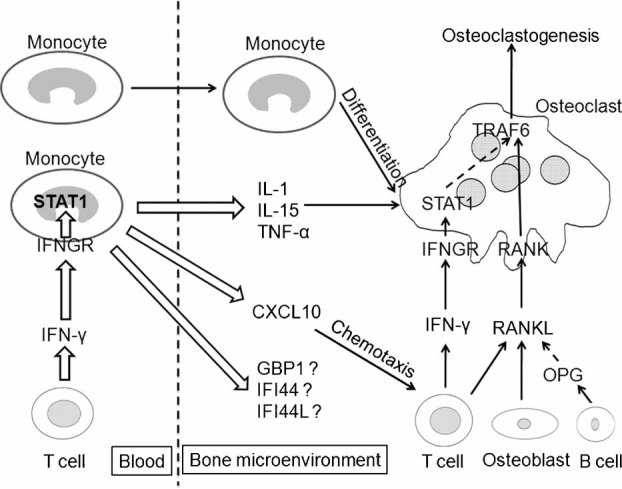
Diagram of the osteoclastogenesis mechanism. The novel pathway mediated by *STAT1* in blood monocytes was connected by big hollow arrows. The solid arrows indicate activation, and the dashed arrows indicate inhibition.

Based on the present results and previous knowledge, we developed a novel mechanism for osteoclastogenesis (Fig. 5). In peripheral blood, IFN mediated by *STAT1* may stimulate circulating monocytes to produce cytokines such as IL-1, TNF, CXCL10, and IL-15 that increase the bone resorption function of osteoclasts. Another pathway in the bone microenvironment also may be triggered by upregulated *STAT1* and IFN, which may inhibit osteoblatogenesis. In this study, no expression of the *RANK* and *TRAF6* genes in circulating monocytes was detected, suggesting that osteoclast formation was completely inhibited in circulating monocytes. The fact that osteoclast differentiation was not initiated in peripheral circulating monocytes in both the high and low BMD groups is possibly because osteoclast formation from monocytes may occur only in a special microenvironment.(52)

Our current research studied and found the importance of only the *STAT1* gene in the function of monocytes or osteoclasts on bone metabolism, which, however, did not address the reported importance of the *STAT1* gene in osteoblasts.(9,44)

In summary, our results support the fact that the *STAT1* gene in circulating monocytes plays important roles in bone metabolism and also suggests that gene expression of the *STAT1*-mediated IFN pathway may be important for osteoporosis.

## Appendix Table

Expression of 168 genes (281 probes) Related to Cytokine, Chemokine, and Osteoclastogenesis-Related Genes in Blood Monocytes From the Low and High BMD Groups

**Table:**

Probe ID	Gene symbol	Gene title	L-BMD intensity	H-BMD intensity	Fold L/H	Raw *P* value	Adjusted *P* value
200887_s_at	STAT1	Signal transducer and activator of transcription 1, 91 kDa	857.21	582.34	1.47	0.00008	0.02248*
202269_x_at	GBP1	Guanylate binding protein 1, interferon-inducible, 67 kDa, guanylate binding protein 1, interferon-inducible, 67 kDa	401.11	245.99	1.63	0.00024	0.03372*
209969_s_at	STAT1	Signal transducer and activator of transcription 1, 91 kDa	181.82	99.56	1.83	0.00043	0.04027*
231577_s_at	GBP1	Guanylate binding protein 1, interferon-inducible, 67 kDa	680	363.24	1.87	0.00075	0.052687
207375_s_at	IL15RA	Interleukin 15 receptor, alpha	94.78	82.73	1.15	0.00156	0.062623
204533_at	CXCL10	chemokine (C-X-C motif) ligand 10	162.43	83.22	1.95	0.00155	0.062623
204533_at	CXCL10	Chemokine (C-X-C motif) ligand 10	162.43	83.22	1.95	0.00155	0.062623
202270_at	GBP1	Guanylate binding protein 1, interferon-inducible, 67 kDa, guanylate binding protein 1, interferon-inducible, 67 kDa	518.93	288	1.8	0.00221	0.077626
205992_s_at	IL15	Interleukin 15	234.54	174.21	1.35	0.0031	0.096789
216598_s_at	CCL2	Chemokine (C-C motif) ligand 2	16.08	8.82	1.82	0.00396	0.11128
222484_s_at	CXCL14	Chemokine (C-X-C motif) ligand 14	2.68	3.6	0.75	0.00503	0.12849
217371_s_at	IL15	Interleukin 15	114.54	88.29	1.3	0.00693	0.1391
214453_s_at	IFI44	Interferon-induced protein 44	321.75	186.31	1.73	0.00693	0.1391
1560791_at	CXCL9	Chemokine (C-X-C motif) ligand 9	8.51	8.08	1.05	0.00692	0.1391
217502_at	IFIT2	Interferon-induced protein with tetratricopeptide repeats 2	104.43	75.69	1.38	0.00746	0.13975
207902_at	IL5RA	Interleukin 5 receptor, alpha	22.33	19.24	1.16	0.00861	0.14628
236897_at	IL17RB	Interleukin 17 receptor, beta	3.45	5.03	0.69	0.00933	0.14628
225636_at	STAT2	Signal transducer and activator of transcription 2, 113 kDa	404.73	339.6	1.19	0.00937	0.14628
202688_at	TNFSF10	Tumor necrosis factor (ligand) superfamily, member 10, tumor necrosis factor (ligand) superfamily, member 10	1094.65	889.05	1.23	0.01084	0.16032
1560999_a_at	IL12RB2	Interleukin 12 receptor, beta 2	7.89	7.96	0.99	0.01247	0.16106
204439_at	IFI44L	Interferon-induced protein 44-like	424.81	153.59	2.77	0.01261	0.16106
205599_at	TRAF1	TNF receptor-associated factor 1	20.8	23.99	0.87	0.01168	0.16106
202748_at	GBP2	Guanylate binding protein 2, interferon-inducible, guanylate binding protein 2, interferon-inducible	307.78	240.71	1.28	0.01354	0.16542
227264_at	TRAF6	TNF receptor-associated factor 6	31.43	31.59	0.99	0.01545	0.18089
242907_at	GBP2	Guanylate binding protein 2, interferon-inducible	335.3	214.65	1.56	0.01677	0.18849
209417_s_at	IFI35	Interferon-induced protein 35	158.99	115.84	1.37	0.02209	0.23874
204747_at	IFIT3	Interferon-induced protein with tetratricopeptide repeats 3	162.68	103.01	1.58	0.0236	0.24561
201642_at	IFNGR2	Interferon gamma receptor 2 (interferon gamma transducer 1)	831.32	811.55	1.02	0.02882	0.27926
214038_at	CCL8	Chemokine (C-C motif) ligand 8	34.53	23.4	1.48	0.02868	0.27926
212657_s_at	IL1RN	Interleukin 1 receptor antagonist	468.82	256.04	1.83	0.03721	0.30425
211517_s_at	IL5RA	Interleukin 5 receptor, alpha	18.71	19.07	0.98	0.03265	0.30425
204863_s_at	IL6ST	Interleukin 6 signal transducer (gp130, oncostatin M receptor)	80.19	57.06	1.41	0.04215	0.30425
206693_at	IL7	Interleukin 7	28.73	32.91	0.87	0.03724	0.30425
206618_at	IL18R1	Interleukin 18 receptor 1	11.01	21.06	0.52	0.04753	0.30425
221658_s_at	IL21R	Interleukin 21 receptor	11.34	13.19	0.86	0.03918	0.30425
237493_at	IL22RA2	Interleukin 22 receptor, alpha 2	10.52	14.41	0.73	0.04177	0.30425
1552912_a_at	IL23R	Interleukin 23 receptor	5.7	5.07	1.12	0.04464	0.30425
206569_at	IL24	Interleukin 24	16.97	15.76	1.08	0.04745	0.30425
207964_x_at	IFNA4	Interferon, alpha 4	15.55	6.72	2.31	0.04468	0.30425
232375_at	STAT1	Signal transducer and activator of transcription 1, 91 kDa	142.43	89.1	1.6	0.04764	0.30425
226757_at	IFIT2	Interferon-induced protein with tetratricopeptide repeats 2	292.64	205.9	1.42	0.03721	0.30425
231578_at	GBP1	Guanylate binding protein 1, interferon-inducible, 67 kDa	20.83	15.71	1.33	0.04753	0.30425
210390_s_at	CCL14, CCL15	Chemokine (C-C motif) ligand 14, chemokine (C-C motif) ligand 15	2.8	2.81	0.99	0.04764	0.30425
210549_s_at	CCL23	Chemokine (C-C motif) ligand 23	9.67	14.94	0.65	0.03958	0.30425
206172_at	IL13RA2	Interleukin 13 receptor, alpha 2	3.4	3.84	0.89	0.05017	0.30837
229263_at	IL17RD	Interleukin 17 receptor D	6.23	5.42	1.15	0.05048	0.30837
207160_at	IL12A	Interleukin 12A (natural killer cell stimulatory factor 1, cytotoxic lymphocyte maturation factor 1, p35)	4.14	4.3	0.96	0.05355	0.31472
202411_at	IFI27	Interferon, alpha-inducible protein 27	32.67	15.61	2.09	0.05376	0.31472
208164_s_at	IL9R	Interleukin 9 receptor	4.71	5.64	0.84	0.05639	0.32338
208375_at	IFNA1	Interferon, alpha 1	2.8	5.44	0.52	0.06403	0.34601
1555464_at	IFIH1	Interferon induced with helicase C domain 1	70.3	50.39	1.4	0.06403	0.34601
1569861_at	TRAF5	TNF receptor-associated factor 5	6.6	7.1	0.93	0.06403	0.34601
209687_at	CXCL12	Chemokine (C-X-C motif) ligand 12 (stromal cell-derived factor 1)	5.19	6.84	0.76	0.07578	0.40178
224283_x_at	IL18BP	Interleukin 18 binding protein	15.36	18.89	0.81	0.08023	0.41749
215561_s_at	IL1R1	Interleukin 1 receptor, type I	5.66	9.41	0.6	0.08451	0.42486
202687_s_at	TNFSF10	Tumor necrosis factor (ligand) superfamily, member 10, tumor necrosis factor (ligand) superfamily, member 10	556.18	440.74	1.26	0.08467	0.42486
234516_at	IL1R1	Interleukin 1 receptor, type I	24.23	26.62	0.91	0.09437	0.43578
212196_at	IL6ST	Interleukin 6 signal transducer (gp130, oncostatin M receptor)	131.01	127.64	1.03	0.0946	0.43578
206926_s_at	IL11	Interleukin 11	11.27	10.34	1.09	0.09268	0.43578
221926_s_at	IL17RC	Interleukin 17 receptor C	4.33	4.36	0.99	0.09392	0.43578
205476_at	CCL20	Chemokine (C-C motif) ligand 20	51.06	9.12	5.6	0.09404	0.43578
210548_at	CCL23	Chemokine (C-C motif) ligand 23	3.53	4.61	0.77	0.09949	0.45091
216243_s_at	IL1RN	Interleukin 1 receptor antagonist	134.21	88.04	1.52	0.10519	0.46185
209827_s_at	IL16	Interleukin 16 (lymphocyte chemoattractant factor)	198.96	253.61	0.78	0.1049	0.46185
211338_at	IFNA2	Interferon, alpha 2	6.31	6.85	0.92	0.1101	0.47597
205099_s_at	CCR1	Chemokine (C-C motif) receptor 1	381.92	370.82	1.03	0.11671	0.4969
205207_at	IL6	Interleukin 6 (interferon, beta 2)	33.83	11.29	3	0.12912	0.49729
204773_at	IL11RA	Interleukin 11 receptor, alpha	35.77	37.85	0.94	0.12892	0.49729
222974_at	IL22	Interleukin 22	6.78	5.04	1.35	0.12906	0.49729
220054_at	IL23A	Interleukin 23, alpha subunit p19	18.45	22.23	0.83	0.1225	0.49729
207113_s_at	TNF	Tumor necrosis factor	411.29	195.36	2.11	0.12276	0.49729
208075_s_at	CCL7	Chemokine (C-C motif) ligand 7, chemokine (C-C motif) ligand 7	5.28	8.89	0.59	0.12892	0.49729
1569203_at	CXCL2	Chemokine (C-X-C motif) ligand 2	36.79	18.62	1.98	0.12919	0.49729
207681_at	CXCR3	Chemokine (C-X-C motif) receptor 3	34.98	46.84	0.75	0.13547	0.51442
39402_at	IL1B	Interleukin 1, beta	1285.79	443.95	2.9	0.14268	0.52501
207906_at	IL3	Interleukin 3 (colony-stimulating factor, multiple)	2.26	2.21	1.02	0.14947	0.52501
208193_at	IL9	Interleukin 9	3.69	3.14	1.17	0.14905	0.52501
206924_at	IL11	Interleukin 11	2.88	3.68	0.78	0.14919	0.52501
235531_at	IL17RB	Interleukin 17 receptor B	20.83	20.61	1.01	0.14234	0.52501
214458_at	TRAF3IP1	TNF receptor-associated factor 3 interacting protein 1	11.98	11.86	1.01	0.14878	0.52501
64440_at	IL17RC	Interleukin 17 receptor C	79.17	86.92	0.91	0.15723	0.52597
229450_at	IFIT3	Interferon-induced protein with tetratricopeptide repeats 3	476.16	293.63	1.62	0.15723	0.52597
209774_x_at	CXCL2	Chemokine (C-X-C motif) ligand 2	601.98	172.21	3.5	0.15723	0.52597
207850_at	CXCL3	Chemokine (C-X-C motif) ligand 3	201.37	80.34	2.51	0.15723	0.52597
202948_at	IL1R1	Interleukin 1 receptor, type I	31.23	37.41	0.83	0.16448	0.53815
212659_s_at	IL1RN	Interleukin 1 receptor antagonist	129.83	117.41	1.11	0.1647	0.53815
211372_s_at	IL1R2	Interleukin 1 receptor, type II	15.5	19.1	0.81	0.17281	0.54583
1552915_at	IL28A	Interleukin 28A (interferon, lambda 2)	21.28	18.3	1.16	0.17288	0.54583
223710_at	CCL26	Chemokine (C-C motif) ligand 26	19.21	17.31	1.11	0.17244	0.54583
205926_at	IL27RA	Interleukin 27 receptor, alpha	142.43	145.56	0.98	0.18105	0.55907
204932_at	TNFRSF11B	Tumor necrosis factor receptor superfamily, member 11b, osteoprotegerin (OGP)	10.14	12.21	0.83	0.18001	0.55907
220971_at	IL17E	Interleukin 17E	10.42	12.41	0.84	0.18921	0.56675
219255_x_at	IL17RB	Interleukin 17 receptor B	6.93	8.36	0.83	0.18853	0.56675
217199_s_at	STAT2	Signal transducer and activator of transcription 2, 113 kDa	31.51	25.83	1.22	0.18959	0.56675
210118_s_at	IL1A	Interleukin 1, alpha	23.56	10.89	2.16	0.21594	0.57929
211516_at	IL5RA	Interleukin 5 receptor, alpha	5.92	8.51	0.69	0.21515	0.57929
202859_x_at	IL8	Interleukin 8	1850.81	730.41	2.53	0.22677	0.57929
209828_s_at	IL16	Interleukin 16 (lymphocyte chemoattractant factor)	23.08	34.55	0.67	0.2267	0.57929
208402_at	IL17	Interleukin 17 (cytotoxic T-lymphocyte-associated serine esterase 8)	14.02	19.57	0.72	0.2059	0.57929
221165_s_at	IL22	Interleukin 22	21.89	23.56	0.93	0.22486	0.57929
221111_at	IL26	Interleukin 26	4.13	5.1	0.81	0.19841	0.57929
225669_at	IFNAR1	Interferon (alpha, beta, and omega) receptor 1	37.99	40.96	0.93	0.22677	0.57929
1552611_a_at	JAK1	Janus kinase 1 (a protein tyrosine kinase)	169.58	162.82	1.04	0.22677	0.57929
201422_at	IFI30	Interferon, gamma-inducible protein 30	3459.22	3226.64	1.07	0.22646	0.57929
204352_at	TRAF5	TNF receptor-associated factor 5	23.86	37.43	0.64	0.20762	0.57929
207900_at	CCL17	Chemokine (C-C motif) ligand 17	20.27	20.66	0.98	0.22662	0.57929
207445_s_at	CCR9	Chemokine (C-C motif) receptor 9	18.78	21.37	0.88	0.21657	0.57929
205242_at	CXCL13	Chemokine (C-X-C motif) ligand 13 (B-cell chemoattractant)	2.43	3.62	0.67	0.1978	0.57929
211469_s_at	CXCR6	Chemokine (C-X-C motif) receptor 6	4.67	6.83	0.68	0.21586	0.57929
203687_at	CX3CL1	Chemokine (C-X3-C motif) ligand 1	4.28	5.34	0.8	0.20715	0.57929
218002_s_at	CXCL14	Chemokine (C-X-C motif) ligand 14	2.03	2.11	0.96	0.23609	0.59767
205067_at	IL1B	Interleukin 1, beta	1436.05	513.56	2.8	0.25774	0.62978
210744_s_at	IL5RA	Interleukin 5 receptor, alpha	7.31	10.81	0.68	0.25733	0.62978
1552609_s_at	IL28A /// IL28B	Interleukin 28A (interferon, lambda 2), interleukin 28B (interferon, lambda 3)	10.3	10.99	0.94	0.25766	0.62978
242473_at	TRAF4	TNF receptor-associated factor 4	8.1	10.37	0.78	0.25774	0.62978
243977_at	IL6	Interleukin 6 (interferon, beta 2)	20.98	24.02	0.87	0.26814	0.64011
204103_at	CCL4	Chemokine (C-C motif) ligand 4	828.94	274.36	3.02	0.2688	0.64011
32128_at	CCL18	Chemokine (C-C motif) ligand 18 (pulmonary and activation-regulated)	13.14	15.52	0.85	0.26863	0.64011
207433_at	IL10	Interleukin 10	6.19	7.02	0.88	0.29161	0.67187
212203_x_at	IFITM3	Interferon induced transmembrane protein 3 (1-8U)	1196.83	954.47	1.25	0.2917	0.67187
211153_s_at	TNFSF11	Tumor necrosis factor (ligand) superfamily, member 11, activator of NF-κB ligand (RANKL)	4.88	4.41	1.11	0.29137	0.67187
207794_at	CCR2	Chemokine (C-C motif) receptor 2, chemokine (C-C motif) receptor 2	224.96	310.1	0.73	0.2917	0.67187
216244_at	IL1RN	Interleukin 1 receptor antagonist	4.14	3.79	1.09	0.31554	0.67755
220056_at	IL22RA1	Interleukin 22 receptor, alpha 1	25.21	21.24	1.19	0.30247	0.67755
208173_at	IFNB1	Interferon, beta 1, fibroblast	3.43	4.31	0.8	0.31521	0.67755
219209_at	IFIH1	Interferon induced with helicase C domain 1	137.64	113.56	1.21	0.31571	0.67755
221571_at	TRAF3	TNF receptor-associated factor 3	36.67	41.08	0.89	0.30354	0.67755
206983_at	CCR6	Chemokine (C-C motif) receptor 6	4.79	7.61	0.63	0.31579	0.67755
208059_at	CCR8	Chemokine (C-C motif) receptor 8	3.42	3.91	0.87	0.31554	0.67755
204470_at	CXCL1	Chemokine (C-X-C motif) ligand 1 (melanoma growth stimulating activity, alpha)	64.18	28.14	2.28	0.31579	0.67755
206336_at	CXCL6	Chemokine (C-X-C motif) ligand 6 (granulocyte chemotactic protein 2)	6.94	9.41	0.74	0.31587	0.67755
207072_at	IL18RAP	Interleukin 18 receptor accessory protein	32.1	70.39	0.46	0.32828	0.68858
216020_at	IFIH1	Interferon induced with helicase C domain 1	10.33	7.56	1.36	0.32836	0.68858
207861_at	CCL22	Chemokine (C-C motif) ligand 22	3.9	4.21	0.93	0.32728	0.68858
207037_at	TNFRSF11A	Tumor necrosis factor receptor superfamily, member 11a, activator of NF-κB, receptor activator of nuclear factor κB (RANK)	12.01	13.75	0.87	0.34092	0.69503
205114_s_at	CCL3, CCL3L1	Chemokine (C-C motif) ligand 3, chemokine (C-C motif) ligand 3-like 1	1514.49	513.72	2.95	0.34133	0.69503
221463_at	CCL24	Chemokine (C-C motif) ligand 24	4.12	7.67	0.54	0.341	0.69503
208304_at	CCR3	Chemokine (C-C motif) receptor 3	55.88	68.99	0.81	0.34133	0.69503
228977_at	IL17D	Interleukin 17D	3.92	4.04	0.97	0.35405	0.69653
205707_at	IL17R	Interleukin 17 receptor	296.73	357.67	0.83	0.35446	0.69653
208261_x_at	IFNA10	Interferon, alpha 10	12.66	14.89	0.85	0.35446	0.69653
208448_x_at	IFNA16	Interferon, alpha 16	20	19.59	1.02	0.35413	0.69653
1562296_at	CXCL14	Chemokine (C-X-C motif) ligand 14	10.64	7.69	1.38	0.35446	0.69653
211506_s_at	IL8	Interleukin 8	1343.34	417.69	3.22	0.36807	0.69884
1552584_at	IL12RB1	Interleukin 12 receptor, beta 1	81.71	88.5	0.92	0.36807	0.69884
208548_at	IFNA6	Interferon, alpha 6	25.55	29.9	0.85	0.36741	0.69884
207354_at	CCL16	Chemokine (C-C motif) ligand 16	7.33	8.09	0.91	0.36766	0.69884
204606_at	CCL21	Chemokine (C-C motif) ligand 21	13.18	9.83	1.34	0.36774	0.69884
224079_at	IL17C	Interleukin 17C	35.95	36.44	0.99	0.39534	0.7044
219115_s_at	IL20RA	Interleukin 20 receptor, alpha	4.77	4.78	1	0.38117	0.7044
219971_at	IL21R	Interleukin 21 receptor	6.05	8.49	0.71	0.39582	0.7044
208344_x_at	IFNA13	Interferon, alpha 13	24.68	14.86	1.66	0.38142	0.7044
203153_at	IFIT1	Interferon-induced protein with tetratricopeptide repeats 1, interferon-induced protein with tetratricopeptide repeats 1	199.61	122.27	1.63	0.39607	0.7044
201601_x_at	IFITM1	Interferon induced transmembrane protein 1 (9-27)	371.78	359.83	1.03	0.39607	0.7044
205558_at	TRAF6	TNF receptor-associated factor 6	40.12	51.7	0.78	0.38101	0.7044
224027_at	CCL28	Chemokine (C-C motif) ligand 28	12.05	12.17	0.99	0.3959	0.7044
203666_at	CXCL12	Chemokine (C-X-C motif) ligand 12 (stromal cell-derived factor 1)	47.91	55.58	0.86	0.39558	0.7044
237038_at	CXCL14	Chemokine (C-X-C motif) ligand 14	4.89	5.25	0.93	0.38044	0.7044
205291_at	IL2RB	Interleukin 2 receptor, beta, interleukin 2 receptor, beta	90.85	134.18	0.68	0.42532	0.7114
206148_at	IL3RA	Interleukin 3 receptor, alpha (low affinity)	33.42	29.59	1.13	0.42516	0.7114
211000_s_at	IL6ST	Interleukin 6 signal transducer (gp130, oncostatin M receptor)	42.99	41.69	1.03	0.42524	0.7114
212195_at	IL6ST	Interleukin 6 signal transducer (gp130, oncostatin M receptor)	333.64	330.73	1.01	0.42532	0.7114
207901_at	IL12B	Interleukin 12B (natural killer cell stimulatory factor 2, cytotoxic lymphocyte maturation factor 2, p40)	13.58	15.13	0.9	0.41006	0.7114
1552610_a_at	JAK1	Janus kinase 1 (a protein tyrosine kinase)	195.54	234.77	0.83	0.42524	0.7114
238494_at	TRAF3IP1	TNF receptor-associated factor 3 interacting protein 1	42.08	48.73	0.86	0.42436	0.7114
206988_at	CCL25	Chemokine (C-C motif) ligand 25	9.38	9.41	1	0.42468	0.7114
224240_s_at	CCL28	Chemokine (C-C motif) ligand 28	23.78	30.34	0.78	0.425	0.7114
1568934_at	CX3CR1	Chemokine (C-X3-C motif) receptor 1	25.24	37.66	0.67	0.425	0.7114
201887_at	IL13RA1	Interleukin 13 receptor, alpha 1	267.86	291.22	0.92	0.45571	0.72771
1561853_a_at	IL23R	Interleukin 23 receptor	4.61	4.55	1.01	0.45463	0.72771
242903_at	IFNGR1	Interferon gamma receptor 1	265.56	251	1.06	0.45579	0.72771
214022_s_at	IFITM1	Interferon induced transmembrane protein 1 (9-27)	593.06	624.52	0.95	0.45579	0.72771
208315_x_at	TRAF3	TNF receptor-associated factor 3	66.38	70.17	0.95	0.4554	0.72771
206978_at	CCR2	Chemokine (C-C motif) receptor 2, chemokine (C-C motif) receptor 2	556.27	705.5	0.79	0.45579	0.72771
207852_at	CXCL5	Chemokine (C-X-C motif) ligand 5	12.5	16.09	0.78	0.45571	0.72771
206974_at	CXCR6	Chemokine (C-X-C motif) receptor 6	10.88	15.49	0.7	0.4554	0.72771
217119_s_at	CXCR3	Chemokine (C-X-C motif) receptor 3	10.84	21.84	0.5	0.4711	0.7479
211405_x_at	IFNA17	Interferon, alpha 17	33.16	36.39	0.91	0.48701	0.76882
217489_s_at	IL6R	Interleukin 6 receptor	35.97	45.58	0.79	0.52027	0.7818
214950_at	IL9R	Interleukin 9 receptor	10.43	21.45	0.49	0.5197	0.7818
201888_s_at	IL13RA1	Interleukin 13 receptor, alpha 1	107.98	114.14	0.95	0.5202	0.7818
219323_s_at	IL18BP	Interleukin 18 binding protein	11.17	18.06	0.62	0.52006	0.7818
1552995_at	IL27	Interleukin 27	7.78	5.66	1.38	0.50336	0.7818
244261_at	IL28RA	Interleukin 28 receptor, alpha (interferon, lambda receptor)	11.39	17.45	0.65	0.52027	0.7818
210643_at	TNFSF11	Tumor necrosis factor (ligand) superfamily, member 11, activator of NF-κB ligand (RANKL)	7.75	11.01	0.7	0.51998	0.7818
206991_s_at	CCR5	Chemokine (C-C motif) receptor 5	53.27	98.45	0.54	0.5202	0.7818
205898_at	CX3CR1	Chemokine (C-X3-C motif) receptor 1	1343.17	1679.71	0.8	0.52027	0.7818
220273_at	IL17B	Interleukin 17B	3.01	6.06	0.5	0.53668	0.79372
224071_at	IL20	Interleukin 20	22.93	24.02	0.95	0.53605	0.79372
1555499_a_at	IL28RA	Interleukin 28 receptor, alpha (interferon, lambda receptor)	23.78	22.54	1.06	0.53668	0.79372
207538_at	IL4	Interleukin 4	9.75	10.11	0.96	0.55412	0.80688
204912_at	IL10RA	Interleukin 10 receptor, alpha	681.13	656.72	1.04	0.55419	0.80688
223030_at	TRAF7	TNF receptor-associated factor 7	13.44	18.42	0.73	0.55405	0.80688
205403_at	IL1R2	Interleukin 1 receptor, type II	36.25	48.67	0.74	0.58915	0.81153
234967_at	IL6ST	Interleukin 6 signal transducer (gp130, oncostatin M receptor)	3.65	3.37	1.08	0.58864	0.81153
1552646_at	IL11RA	Interleukin 11 receptor, alpha	46.73	58.54	0.8	0.56956	0.81153
222062_at	IL27RA	Interleukin 27 receptor, alpha	141.68	184.7	0.77	0.58915	0.81153
204191_at	IFNAR1	Interferon (alpha, beta, and omega) receptor 1	28.97	25.98	1.12	0.58639	0.81153
206332_s_at	IFI16	Interferon, gamma-inducible protein 16	742.15	756.26	0.98	0.58915	0.81153
211899_s_at	TRAF4	TNF receptor-associated factor 4	8.43	9.49	0.89	0.57062	0.81153
223029_s_at	TRAF7	TNF receptor-associated factor 7	39.65	31.97	1.24	0.57141	0.81153
210072_at	CCL19	Chemokine (C-C motif) ligand 19	20.58	19.63	1.05	0.58896	0.81153
207955_at	CCL27	Chemokine (C-C motif) ligand 27	29.49	33.85	0.87	0.5889	0.81153
211122_s_at	CXCL11	Chemokine (C-X-C motif) ligand 11	17.69	8.56	2.07	0.57121	0.81153
205098_at	CCR1	Chemokine (C-C motif) receptor 1	514.24	489.35	1.05	0.60695	0.83197
204116_at	IL2RG	Interleukin 2 receptor, gamma (severe combined immunodeficiency)	193.16	255.56	0.76	0.62505	0.83249
205945_at	IL6R	Interleukin 6 receptor, interleukin 6 receptor	258.64	261.18	0.99	0.62505	0.83249
205798_at	IL7R	Interleukin 7 receptor, interleukin 7 receptor	64.86	104.79	0.62	0.62511	0.83249
206295_at	IL18	Interleukin 18 (interferon-γ-inducing factor)	67.16	76.06	0.88	0.62511	0.83249
208965_s_at	IFI16	Interferon, gamma-inducible protein 16	347.81	327.78	1.06	0.62511	0.83249
1555759_a_at	CCL5	Chemokine (C-C motif) ligand 5	972.23	900.04	1.08	0.62511	0.83249
204864_s_at	IL6ST	Interleukin 6 signal transducer (gp130, oncostatin M receptor)	29.53	30.2	0.98	0.66186	0.84938
221947_at	IL17RC	Interleukin 17 receptor C	47.88	44.71	1.07	0.66175	0.84938
217702_at	IL27RA	Interleukin 27 receptor, alpha	14.93	12.41	1.2	0.64337	0.84938
214569_at	IFNA5	Interferon, alpha 5	6.93	6.7	1.03	0.66083	0.84938
1553574_at	IFNE1	Interferon epsilon 1	15.13	14.11	1.07	0.66099	0.84938
201648_at	JAK1	Janus kinase 1 (a protein tyrosine kinase)	543.47	603.1	0.9	0.66197	0.84938
204413_at	TRAF2	TNF receptor-associated factor 2	3.8	4.76	0.8	0.6617	0.84938
214974_x_at	CXCL5	Chemokine (C-X-C motif) ligand 5	529.1	524.14	1.01	0.66197	0.84938
207849_at	IL2	Interleukin 2	6.56	6.19	1.06	0.68047	0.85388
208259_x_at	IFNA7	Interferon, alpha 7	26.13	17.84	1.46	0.68057	0.85388
208182_x_at	IFNA14	Interferon, alpha 14	27.87	34.38	0.81	0.67964	0.85388
202727_s_at	IFNGR1	Interferon gamma receptor 1	1046.25	1094.15	0.96	0.68067	0.85388
210163_at	CXCL11	Chemokine (C-X-C motif) ligand 11	17.34	8.8	1.97	0.68041	0.85388
226333_at	IL6R	Interleukin 6 receptor	370.68	448.76	0.83	0.69968	0.86232
1555016_at	IL16	Interleukin 16 (lymphocyte chemoattractant factor)	37.26	36.32	1.03	0.69914	0.86232
204933_s_at	TNFRSF11B	Tumor necrosis factor receptor superfamily, member 11b, osteoprotegerin (OGP)	6.46	4.64	1.39	0.69968	0.86232
206337_at	CCR7	Chemokine (C-C motif) receptor 7, chemokine (C-C motif) receptor 7	57.88	65.73	0.88	0.69968	0.86232
207008_at	IL8RB	Interleukin 8 receptor, beta	179.1	225.48	0.79	0.71877	0.87815
206890_at	IL12RB1	Interleukin 12 receptor, beta 1	34.16	45.32	0.75	0.71877	0.87815
206999_at	IL12RB2	Interleukin 12 receptor, beta 2	15.38	18.64	0.83	0.73792	0.88263
234408_at	IL17F	Interleukin 17F	2.32	2.44	0.95	0.73753	0.88263
201315_x_at	IFITM2	Interferon induced transmembrane protein 2 (1-8D)	1306.87	1373.99	0.95	0.73814	0.88263
205392_s_at	CCL14, / CCL15	Chemokine (C-C motif) ligand 14, chemokine (C-C motif) ligand 15	12.36	15.56	0.79	0.73722	0.88263
217028_at	CXCR4	Chemokine (C-X-C motif) receptor 4	438.44	468.81	0.94	0.73814	0.88263
211269_s_at	IL2RA	Interleukin 2 receptor, alpha	24.39	26.68	0.91	0.75738	0.89052
221271_at	IL21	Interleukin 21	6.93	7	0.99	0.75726	0.89052
209924_at	CCL18	Chemokine (C-C motif) ligand 18 (pulmonary and activation-regulated)	18.18	16.69	1.09	0.75726	0.89052
203915_at	CXCL9	Chemokine (C-X-C motif) ligand 9	42.84	36.42	1.18	0.75742	0.89052
216876_s_at	IL17	Interleukin 17 (cytotoxic T-lymphocyte-associated serine esterase 8)	2.99	2.74	1.09	0.77715	0.90614
235116_at	TRAF1	TNF receptor-associated factor 1	26.19	31.3	0.84	0.7767	0.90614
210133_at	CCL11	Chemokine (C-C motif) ligand 11	13.59	17.24	0.79	0.79698	0.92161
208376_at	CCR4	Chemokine (C-C motif) receptor 4	21.06	20.58	1.02	0.79671	0.92161
217212_s_at	IL9R	Interleukin 9 receptor	55.78	61.46	0.91	0.8169	0.92938
214059_at	IFI44	Interferon-induced protein 44	67.79	53.63	1.26	0.81693	0.92938
230327_at	CCL27	Chemokine (C-C motif) ligand 27	17.48	15.39	1.14	0.81681	0.92938
823_at	CX3CL1	Chemokine (C-X3-C motif) ligand 1	21.62	19.67	1.1	0.81681	0.92938
207539_s_at	IL4	Interleukin 4	19.68	18.36	1.07	0.85704	0.94123
203233_at	IL4R	Interleukin 4 receptor	120.73	114.06	1.06	0.85714	0.94123
209575_at	IL10RB	Interleukin 10 receptor, beta	279.27	295.77	0.94	0.89769	0.94123
211612_s_at	IL13RA1	Interleukin 13 receptor, alpha 1, interleukin 13 receptor, alpha 1	150.14	153.82	0.98	0.89762	0.94123
227401_at	IL17D	Interleukin 17 receptor D	8.6	7.62	1.13	0.89758	0.94123
224156_x_at	IL17RB	Interleukin 17 receptor B	18.08	13.81	1.31	0.87718	0.94123
224361_s_at	IL17RB	Interleukin 17 receptor B	10.48	16.9	0.62	0.89753	0.94123
227997_at	IL17RD	Interleukin 17 receptor D	27.27	32.84	0.83	0.89762	0.94123
236186_x_at	IL17RE	Interleukin 17 receptor E	2.38	3.13	0.76	0.85702	0.94123
222868_s_at	IL18BP	Interleukin 18 binding protein	56.12	58.32	0.96	0.87729	0.94123
222829_s_at	IL20RA	Interleukin 20 receptor, alpha	4.78	3.86	1.24	0.87731	0.94123
1552917_at	IL29	Interleukin 29 (interferon, lambda 1)	33.83	37.22	0.91	0.89769	0.94123
204786_s_at	IFNAR2	Interferon (alpha, beta, and omega) receptor 2	124.38	130.38	0.95	0.89765	0.94123
211676_s_at	IFNGR1	Interferon gamma receptor 1, interferon gamma receptor 1	479.53	517.04	0.93	0.85714	0.94123
205170_at	STAT2	Signal transducer and activator of transcription 2, 113 kDa	47.35	46.01	1.03	0.83697	0.94123
208966_x_at	IFI16	Interferon, gamma-inducible protein 16	591.69	599.19	0.99	0.85714	0.94123
238846_at	TNFRSF11A	Tumor necrosis factor receptor superfamily, member 11a, activator of NF-κB, receptor activator of nuclear factor κB (RANK)	18.53	19.76	0.94	0.85694	0.94123
207533_at	CCL1	Chemokine (C-C motif) ligand 1	2.38	3.42	0.69	0.8976	0.94123
206407_s_at	CCL13	Chemokine (C-C motif) ligand 13	3.59	6.21	0.58	0.83686	0.94123
215101_s_at	CXCL5	Chemokine (C-X-C motif) ligand 5	95.13	96.57	0.99	0.89769	0.94123
211919_s_at	CXCR4	Chemokine (C-X-C motif) receptor 4, chemokine (C-X-C motif) receptor 4	318.92	339.41	0.94	0.89767	0.94123
209201_x_at	CXCR4	Chemokine (C-X-C motif) receptor 4	305.74	310.98	0.98	0.91805	0.959
210904_s_at	IL13RA1	Interleukin 13 receptor, alpha 1	117.07	128.58	0.91	0.9385	0.97313
1405_i_at	CCL5	Chemokine (C-C motif) ligand 5	1312.38	1268.12	1.03	0.9385	0.97313
202871_at	TRAF4	TNF receptor-associated factor 4	18.68	17.18	1.09	0.95897	0.98707
223454_at	CXCL16	Chemokine (C-X-C motif) ligand 16	108.07	104.49	1.03	0.95894	0.98707
207952_at	IL5	Interleukin 5 (colony-stimulating factor, eosinophil)	10.71	13.63	0.79	0.97945	0.99721
207844_at	IL13	Interleukin 13	18.97	32.38	0.59	0.97947	0.99721
224514_x_at	IL17RC	Interleukin 17 receptor C, interleukin 17 receptor C	29.28	36.3	0.81	0.97944	0.99721
206341_at	IL2RA	Interleukin 2 receptor, alpha	12.05	15.51	0.78	1	1
226218_at	IL7R	Interleukin 7 receptor	62.02	108.45	0.57	1	1
220745_at	IL19	Interleukin 19	25.66	29.86	0.86	1	1
225661_at	IFNAR1	Interferon (alpha, beta, and omega) receptor 1	76.46	82.39	0.93	1	1
204785_x_at	IFNAR2	Interferon (alpha, beta, and omega) receptor 2	116.43	124.34	0.94	1	1

*Note:* The data were sorted by adjusted *P* value. Hybridized intensity and “present” status were given based on the MAS5 algorithm. L-BMD intensity means average hybridized intensity in the low BMD group; H-BMD intensity means average hybridized intensity in the high BMD group; Fold L/H means the ration of L-BMD intensity to H-BMD intensity. Raw *P* value represents *P* value before multiple testing correction. Adjusted *P* value represents *P* value adjusted with Benjamini/Hochberg method; asterisks indicate significant results after Benjamini-Hochberg correction considering 281 probes of selected genes.
